# Transposable elements become active and mobile in the genomes of aging mammalian somatic tissues

**DOI:** 10.18632/aging.100621

**Published:** 2013-12-07

**Authors:** Marco De Cecco, Steven W. Criscione, Abigail L. Peterson, Nicola Neretti, John M. Sedivy, Jill A. Kreiling

**Affiliations:** Department of Molecular Biology, Cell Biology and Biochemistry, Center for Genomics and Proteomics, Brown University, Providence, RI 02903, USA

**Keywords:** Aging, epigenetics, chromatin, transposable elements

## Abstract

Transposable elements (TEs) were discovered by Barbara McClintock in maize and have since been found to be ubiquitous in all living organisms. Transposition is mutagenic and organisms have evolved mechanisms to repress the activity of their endogenous TEs. Transposition in somatic cells is very low, but recent evidence suggests that it may be derepressed in some cases, such as cancer development. We have found that during normal aging several families of retrotransposable elements (RTEs) start being transcribed in mouse tissues. In advanced age the expression culminates in active transposition. These processes are counteracted by calorie restriction (CR), an intervention that slows down aging. Retrotransposition is also activated in age-associated, naturally occurring cancers in the mouse. We suggest that somatic retrotransposition is a hitherto unappreciated aging process. Mobilization of RTEs is likely to be an important contributor to the progressive dysfunction of aging cells.

## INTRODUCTION

Aging is the primary risk factor for multiple diseases that are responsible for considerable morbidity and mortality, including dementias, cardiovascular diseases, diabetes and cancer. While aging is clearly multi-factorial, an important driver is believed to be the accumulation of DNA damage and epigenetic changes that lead to genome instability over time, especially in disorders such as cancer [1]. Genome integrity is maintained by multiple pathways involving the DNA damage response machinery and chromatin remodeling complexes. These pathways, which are normally highly efficient, begin to lose their effectiveness with age contributing to destabilization of the genome [2-4]. Multiple environmental as well as endogenous sources of DNA damage have been documented. One potentially important mechanism impacting genome stability is the activation of endogenous transposable elements (TEs), which can result in insertional mutagenesis, DNA damage and genome rearrangements [5].

Approximately half of mammalian genomes are comprised of repetitive sequences [6]. Among the several classes of repetitive sequences, the two most abundant are non-coding tandem repeats (centromeric satellites, telomeric repeats) and TEs. Over evolutionary time most TEs have acquired multiple mutations and are no longer active, however a small fraction retain the ability to transpose [7,8]. The most prominent TEs in the mammalian genome are retrotransposable elements (RTEs), which transpose through an RNA intermediate that is reverse transcribed into DNA and inserted at new sites in the genome [9]. There are three major families of RTEs: the long terminal repeat (LTR) RTEs, which include retroviruses, the long interspersed nuclear elements (LINEs), and the short interspersed nuclear elements (SINEs). LTR RTEs and LINEs are autonomous by virtue of encoding reverse transcriptase and other proteins required for the retrotransposition process, whereas SINEs are non-coding and exploit the machinery encoded by LINEs to transpose.

Because of their autonomous replication, RTEs are commonly viewed as molecular parasites of the genomes that they inhabit [10]. The role that RTEs play in the physiology as well as evolution of their host organisms has been widely debated. On the one hand, transposition can promote evolution by promoting genetic diversity, for example by creating new patterns of gene expression through processes such as promoter recruitment or alternative splicing [11]. Conversely, transposition is often profoundly deleterious, leading to insertional mutagenesis, DNA damage and genome instability [12-14]. Excess or uncontrolled transposition is clearly deleterious, and several mechanisms by which cells can keep RTEs tightly repressed have been documented [15]. The first line of defense is heterochromatinization of the elements in the genome to prevent their transcription. Expression of RTEs is also opposed by several small noncoding RNA mechanisms.

Much of what is known about RTEs reflects their activity in the germline, since transposition events in somatic tissues would not be inherited from generation to generation [16]. Until recently retrotransposition in the soma was thought to be quite rare. However, recent evidence indicates that it may occur much more frequently than previously believed, especially during early embryogenesis. One tissue where retrotransposition appears to be especially active is the nervous system: L1 mobilization was observed in neural progenitor cells during development as well as in adult neurogenesis [17,18], and frequent new L1 insertions were reported in adult brain tissue relative to other tissues [19]. Another group, however, found very few insertions [20]. One pathological process that appears to frequently lead to RTE activation is the development of cancer [21-24].

We recently reported that RTEs become derepressed and start actively transposing during replicative senescence of human fibroblasts in cell culture [25]. In this communication we extend these studies to the investigation of aging mouse tissues.

## RESULTS

### Aging mouse tissues display decreased sensitivity to nuclease digestion

**Figure 1:**
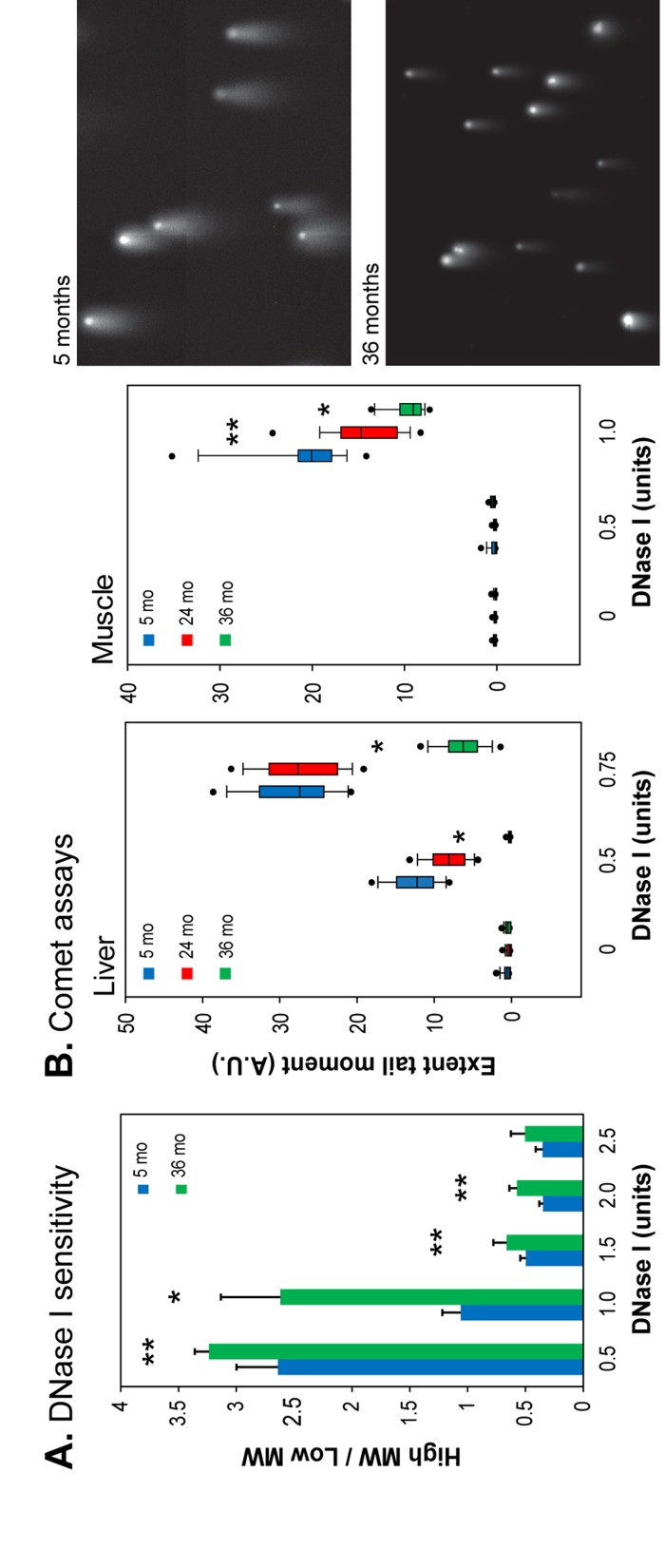
DNase I sensitivity of chromatin in intact nuclei (**A**) The ratio of high to low molecular weight DNA for liver nuclei was calculated following agarose gel electrophoresis (Supplemental Figure 1). Means and standard deviations are shown. (*) p<0.01, (**) p≤0.05, (see Supplemental Information; mo, months). (**B**) Left panels: the extent tail moment was calculated following single cell electrophoresis (see Supplemental Figure 2 for alternative quantification). Data are shown as box plots (box shows median 50% (25%-75%), whiskers the 90%-10% range, dots the 95% and 5% values (A.U., arbitrary units). Right panels: representative images of Comet assays (liver, 0.5 units DNase I, 10 min).

Accumulating evidence indicates that chromatin structure and organization change profoundly during aging [4]. We found that several marks of heterochromatin become more abundant in nuclei of human diploid fibroblasts (HDFs) undergoing replicative senescence in vitro, as well as in several mouse and primate tissues in vivo [26]. To investigate the functional consequences of these changes we performed nuclease (DNase I) sensitivity assays, a well-established method to assess chromatin compaction in intact nuclei. Nuclei isolated from young (5 month) and old (36 month) mouse livers were incubated with increasing concentrations of DNase I. The resulting DNA size distributions revealed that the ratio of high to low molecular weight DNA was higher in the 36 month old liver nuclei at all DNase I concentrations, indicating that a higher proportion of DNA is resistant to nuclease digestion in this tissue (Figure 1A, Supplemental Figure 1).

To further analyze the extent of DNase I digestion we used single cell electrophoresis (also known as the Comet assay) to visualize the digested DNA. Nuclei isolated from young liver showed greater DNase I digestion, indicated by longer DNA “tails” (and larger extent tail moments) following electrophoresis, than liver nuclei from old animals (Figure 1B, Supplemental Figure 2A). These results show that liver chromatin in young mice has more genomic regions that are accessible to DNase I digestion. Since heterochromatin is more compact, and more resistant to nuclease digestion, these results indicate that a greater proportion of chromatin is in a heterochromatic state in the liver of old animals. Overall, these experiments suggest that chromatin rearranges with age in the liver, causing previously open regions of the genome to become more heterochromatic.

Skeletal muscle nuclei showed the greatest increase in heterochromatin-associated proteins with age, in both mouse and baboon [26]. To determine if skeletal muscle undergoes a similar decrease in chromatin accessibility with age as seen in the liver, nuclei isolated from young (5 month), old (24 month), and very old (36 month) skeletal muscle were subjected it to DNase I digestion and analyzed using the Comet assay. As seen in liver, skeletal muscle nuclei showed increased resistance to DNase I digestion with age, as indicated by the smaller extent tail moment. The 36 month old animals displayed the highest degree of resistance to DNase I (Figure 1B, Supplemental Figure 2B). These results indicate that age-associated chromatin rearrangements, leading to a more nuclease resistant (and presumably more compact) state, occur also in terminally differentiated, postmitotic tissues.

### mRNA expression decreases with age

**Figure 2:**
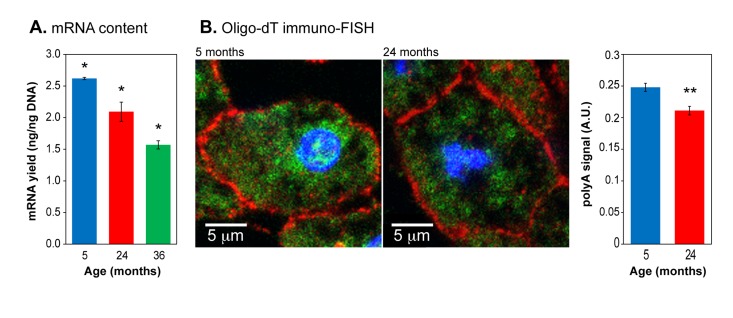
Total mRNA expression in aging liver (**A**) mRNA content in liver tissue was quantified by purification with oligo-dT magnetic beads and normalized to input DNA. (**B**) Left panels: representative images of oligo-dT immuno-FISH staining of liver sections. Nuclei were stained with DAPI (blue). E-cadherin staining (red) highlights cellular membranes. Intracellular mRNA was detected using Cy3-oligo-dT probes (green). Right panel: quantification of polyA^+^ signal. Means and standard deviations are shown. (*) p<0.01; (**) p≤0.05.

An overall increase in heterochromatin should be reflected by a commensurate decrease in RNA production. We therefore quantified the amount of mRNA in liver tissue of mice of varying ages. Using a biochemical purification based on polyA selection we found a decrease of 20% at 24 months which increased to almost 2-fold (42%) at 36 months (Figure 2A). To confirm that these results were not affected by systematic changes during the extraction procedure, we used a different method of mRNA quantification, fluorescence in situ hybridization (FISH) with oligo-dT probes (Figure 2B, Supplemental Figure 3). We found a decrease of 10% at 24 months. These data were acquired by imaging large numbers of nuclei and are statistically significant. It is well known that patterns of gene expression change profoundly with age in a complex and tissue-dependent manner, with both upregulation and downregulation taking place at the individual gene level. The results presented here do not contradict these observations, but in addition indicate that overall steady-state mRNA pools decrease with age, an effect that becomes especially pronounced at very advanced age. To our knowledge this age-associated trend has not been noted before, and agrees closely with the increasing hetero-chromatinization.

### Expression of RTEs and satellite sequences increases with age

**Figure 3:**
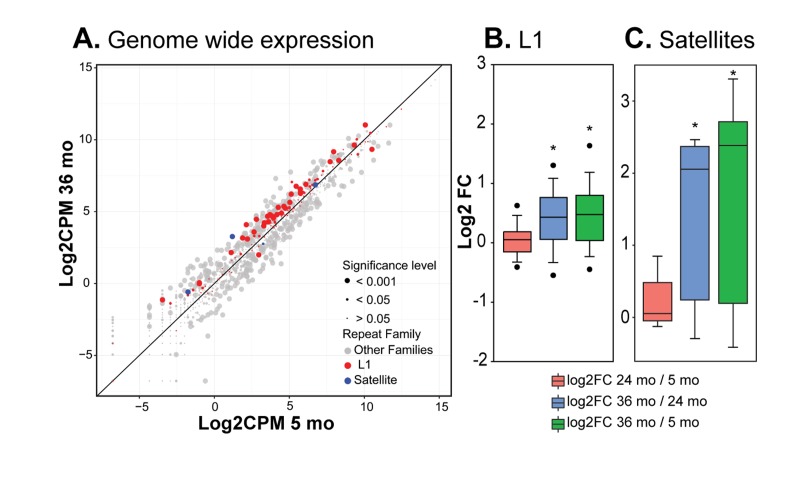
RNA-seq analysis of repetitive element RNA expression (**A**) RNA-seq data from liver of 5, 24 and 36 month old mice (3 per group) were processed with RepEnrich software to estimate the representation of sequence tags mapping to repetitive elements. Normalized log2 counts per million values for 5 versus 36 month animals were plotted for all Repeatmasker annotated repetitive element families (see Supplemental Figure 4 for additional comparisons). Each point represents a sub-family; points above the diagonal are enriched in old animals. Point size indicates FDR-corrected significance levels. L1 and SE sub-families are highlighted (CPM, counts per million). (**B**) The log2 fold changes for the 118 L1 sub-families annotated in the mouse were compared pairwise between age groups (FC, fold change). (**C**) As in B, but comparing the 7 SE sub-families annotated in the mouse. (*) p<0.01. See Methods for details of analysis.

HDF passaged to replicative senescence in cell culture also show an overall trend of increasing hetero-chromatin and decreasing gene activity, but in addition display more complex changes such as relaxation of constitutive heterochromatin in gene poor regions and centromeres [25]. The latter changes allow the transcription of RTEs that culminate in active transposition. To investigate whether these phenomena also occur in mammalian tissues *in vivo* we performed RNA-seq on livers of 5, 24 and 36 month old mice. We analyzed the transcription of L1 LINEs, the largest family of potentially active RTEs, which were also prominently activated in HDF, using a bioinformatic pipeline we recently developed for the analysis of repetitive sequences in high throughput DNA sequencing data [27]. There are 118 L1 subfamilies annotated in the mouse genome (build mm9, www.repeatmasker.org/) and we found that many of them showed increased expression with age (Figure 3A, B). The increases in L1 RNA expression primarily occurred between 24 and 36 months (Supplemental Figure 4A, B).

Satellite elements (SEs) consist of seven different subfamilies of repetitive sequences in the mouse. SEs, which do not transpose, are localized mostly in the centromeric, pericentromeric and telomeric regions of the genome comprising a significant fraction of cellular constitutive heterochromatin. Even though SEs are in a repressed state, they are transcribed at low levels during development, differentiation and in response to stress, and the transcripts may be an important component of kinetochore assembly [28]. SEs became prominently derepressed in HDF after entry into senescence [25]. The RNA-seq analysis showed that likewise the majority of SE subfamilies became derepressed in mouse liver, with the largest changes occurring between 24 and 36 months (Figure 3C).

**Figure 4:**
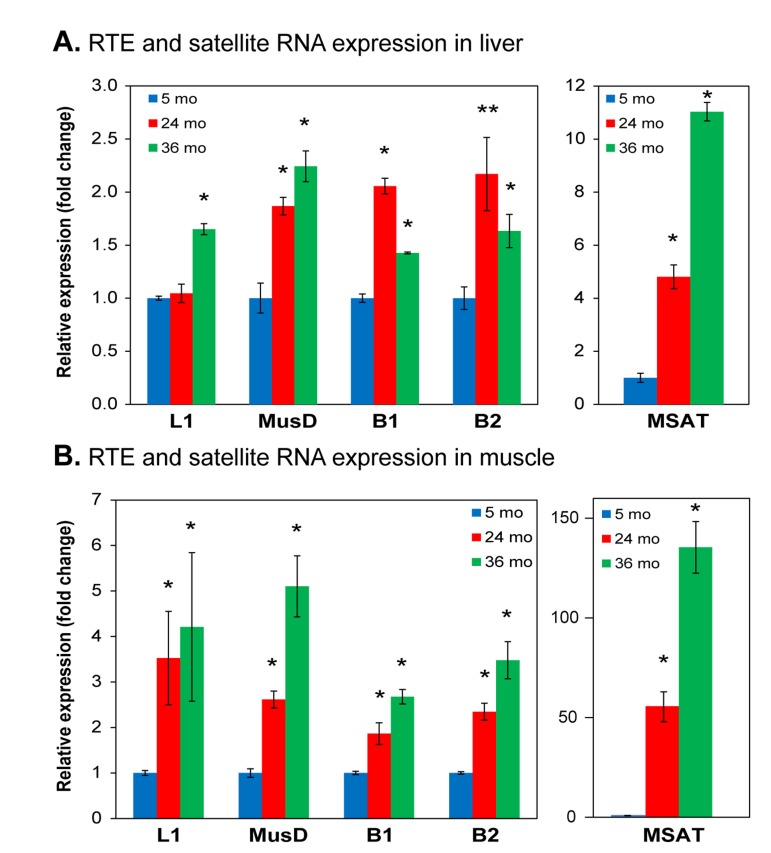
qPCR analysis of RNA expression of representative RTEs and SEs Total RNA was extracted from (**A**) liver and (**B**) skeletal muscle, quantified by qPCR using indicated primers (Table S1) and normalized to GAPDH. Data were additionally normalized to the 5 month value for each element (shown as 1.0). L1, LINE L1; MusD, LTR RTE MusD/ETn; B1, SINE B1; B2, SINE B2; MSAT, major (also known as γ) SE. (*) p<0.01; (**) p≤0.05.

To reinforce and extend these bioinformatic data, as we had done in our studies of HDF [25], we turned to direct quantitative RT-PCR analysis (qPCR) of representative RTE subfamilies of interest. While in humans the only RTEs that are autonomously active are the L1 LINEs, several subfamilies of LTR RTEs are active in the mouse genome [29]. Hence, in addition to L1s, which are the most abundant active RTEs, we investigated the LTR RTEs MusD/ETn, which our RNA-seq data indicated were activated with age. SINEs (such as Alu in humans) are non-autonomous, but can be mobilized by L1 in trans. Since SINE transcripts are not polyadenylated, and hence were not well represented in our RNA-seq dataset, we also examined B1 and B2, the two major SINE families in the mouse genome. Finally, we also assessed the major pericentromeric SEs MSAT. In liver L1 RNA was unchanged at 24 months and showed a statistically significant increase at 36 months (Figure 4A). MusD, B1 and B2 showed an increase at 24 months and either a further increase (MusD) or small drop (B1, B2) at 36 months. MSAT showed a strong increase (5-fold) at 24 months and a further increase (11-fold) at 36 months. Using the same primers we found even more pronounced increases in muscle (Figure 4B), where in all cases RNA expression increased at 24 months with further increases at 36 months (MSAT showed a remarkable 90-fold increase at 36 months).

### Calorie restriction attenuates increased expression of repetitive elements

**Figure 5:**
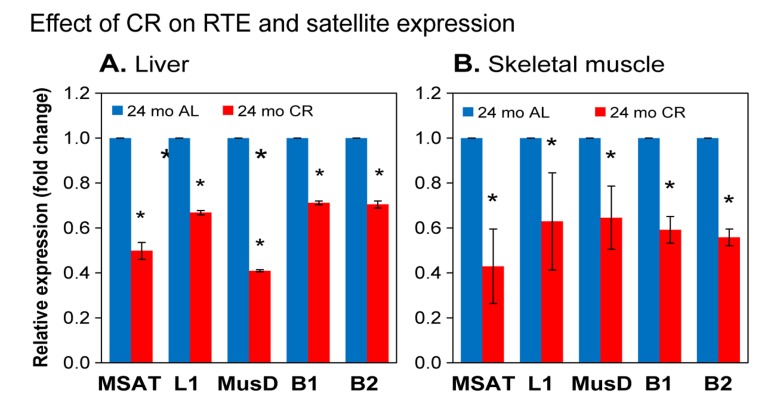
qPCR analysis of RTE and SE RNA expression in calorie restricted (CR) animals (**A**) liver; (**B**) skeletal muscle. Total RNA was extracted from tissues of CR animals and matched *ad libitum* fed (AL) controls. Experiments were performed, analyzed and presented as in Figure 4. 5 CR and 5 age-matched AL animals were used per group. Data were normalized to the 24 month AL value for each element (shown as 1.0). Means and standard deviations are shown. All pairwise comparisons were significant at p≤0.01 (*).

CR is a dietary intervention known to increase lifespan in a wide range of organisms from invertebrates to mice [30]. It is accomplished by the reduction of caloric intake to approximately 60% of a healthy diet without malnutrition. Even though it was recently reported that the ability of CR to increase the lifespan of non-human primates is somewhat ambiguous, it was shown to significantly increase the healthspan (the proportion of lifespan free of age-associated diseases and frailty) of these animals [31,32]. We compared RTE expression in liver and skeletal muscle from *ad libitum* fed (AL) and CR mice at 24 months of age, and found that for all elements examined the RNA levels were significantly decreased in CR animals (Figure 5). We also examined the effect of CR on the transcription pericentromeric SEs. As seen with the RTEs, the upregulation of their transcription was attenuated in 24 month CR animals (Figure 5), indicating the calorie restriction also delays the loss of constitutive hetero-chromatin in centromeric regions. Hence, chromatin reorganization and activation of RTEs are not simply the result of stochastic events that correlate with chronological time, but are causally affected by mechanisms that regulate the rate of aging.

### L1 and MusD copy number increases with age indicating active transposition

**Figure 6:**
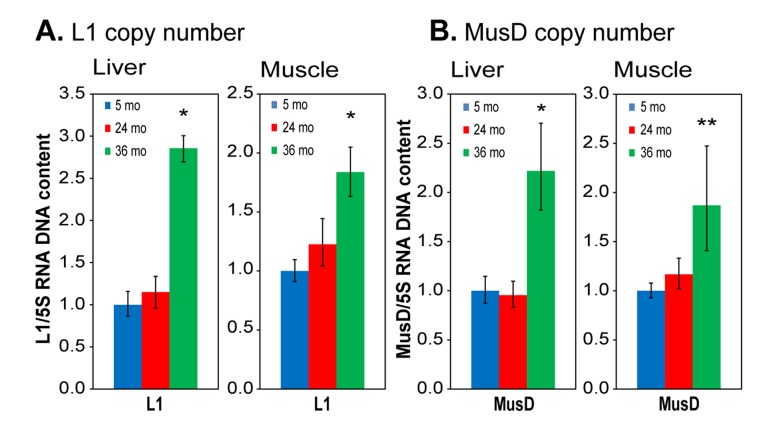
qPCR analysis of DNA to assess RTE genome copy number (**A**) L1; (**B**) MusD. Total DNA was extracted from tissues of the same animals and tissues as used in Figure 4. Relative copy numbers were quantified using a multiplex TaqMan qPCR assay with the indicated primers (Table S1) and normalized to 5S ribosomal DNA. Data were additionally normalized to the 5 month value for each element (shown as 1.0). 5S DNA copy number was independently verified not to vary with age or between animals or tissues using qPCR against known single copy sequences. Means and standard deviations are shown. (*) p<0.01; (**) p≤0.05.

Our data above show that L1 and MusD RNA expression increases with age in liver and skeletal tissues of the mouse. However, RNA expression is only the first step that may eventually lead to transposition, and several cellular defense mechanisms are known to be active downstream of RTE heterochromatinization. Hence, we used the same primers as in the RNA analysis in a sensitive multiplex qPCR assay of genomic DNA [25] to quantify the relative copy numbers of L1 and MusD elements in the genome. As noted above, L1 expression was not increased in the liver of 24 month animals (Figure 4A), and as expected, we did not see an increase in L1 copy number at this age (Figure 6A). We found however a significant increase in L1 expression at 36 months, and we also detected a strong increase in genomic L1 copy number at this age.

In skeletal muscle L1 expression was increased already at 24 months (> 3-fold) and remained high at 36 months of age (Figure 4B). In this case we did not see an increase in copy number at 24 months (only a trend that was not statistically significant), but by 36 months there was a clear increase in copy number (Figure 6B). We do not know the reason for this difference between the two tissues, but a delay between increased expression and transposition is not surprising. Not all L1 transcripts lead to new insertions, as the transposition process is inefficient and often leads to abortive events [13,33]. Furthermore, additional barriers to retrotransposition, such as surveillance by the small interfering RNA pathways, likely require time to overcome. These mechanisms may also differ from one tissue to another. MusD is a LTR retrotransposon with approximately 100 copies in the mouse genome, of which three are believed to be active. MusD expression increased at 24 months in both liver and skeletal muscle (Figure 4), but again without a notable increase in copy number at this age (Figure 6). At 36 months MusD expression increased further, and a significant increase in genome copy number was detectable at this time. These results are consistent with the delay we observed between RNA expression and transposition of L1 RTEs. As with L1, abortive transposition attempts could lead to double stranded breaks that affect overall genome stability.

### L1 and MusD copy numbers increase in naturally occurring, age-associated cancers

**Figure 7:**
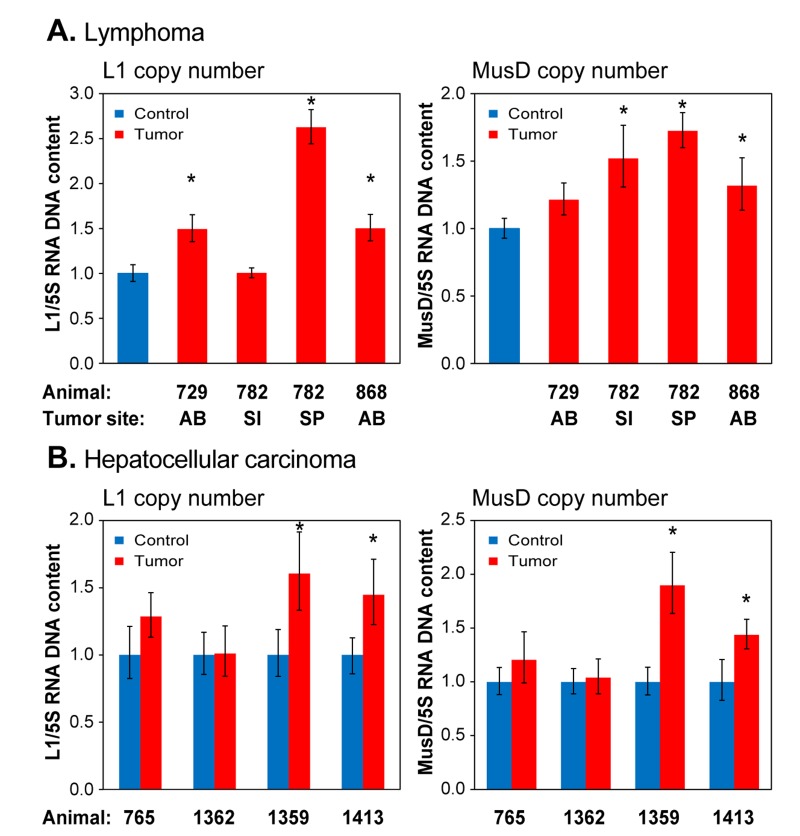
qPCR analysis of DNA to assess RTE genome copy number in spontaneously occurring tumors (**A**) Lymphoma; (**B**) Hepatocellular carcinoma. Analysis was performed as in Figure 6, except that total DNA was extracted from formalin preserved tissue biopsies (see Methods). Animals are designated by their unique identifier numbers. In (**A**) the control was normal liver tissue of an animal of comparable age. AB, abdomen; SI, small intestine; SP, spleen. In (B) the controls were normal tissues cut from the vicinity of the tumor. Means and standard deviations are shown. (*) p<0.01.

Novel transposition events have recently been documented in several human cancers [34]. We therefore examined naturally occurring tumors, which in the C57 strain of mice are primarily lymphomas and hepatocellular carcinomas (HCC), for evidence of RTE mobilization. We found widespread increases in copy number of both L1 and MusD elements, in both lymphoma (Figure 7A) and HCC (Figure 7B). Not all animals showed copy number increases in their cancers, and L1 and MusD copy numbers did not always increase in concert. In comparison to the normal age-associated increases in transposition in the liver (Figure 6), as well as relative to uninvolved tissue in the same animal, it is clear that mobilization of RTEs is significantly increased in HCC.

## DISCUSSION

The inability to maintain complex biological structures contributes to the onset of tissue dysfunction and the eventual demise of organisms as they age. During replicative senescence of human fibroblasts chromatin is subject to extensive changes in the global distribution of euchromatin and heterochromatin [25,35]. We found that the fundamental architecture of the genome undergoes profound alterations: an overall closing of chromatin in euchromatic gene-rich regions, which is opposed by a somewhat paradoxical relaxation of heterochromatin in gene poor and pericentromeric regions [25]. The gene-poor regions overlap significantly with lamin-associated domains (LADs), are marked by H3K9Me3, and replicate late during S phase [35]. The relative closing of euchromatic regions was associated with a dampening of global gene expression, and the relaxation of heterochromatic regions with increased transcription of RTEs, many of which are localized in LADs and are normally heavily heterochromatinized to prevent their expression.

It should be noted that the total level of heterochromatin in the nucleus may increase in senescence, at least as evidenced by increased levels of heterochromatin-associated proteins such as HP1 and the histone variant macro H2A [26]. Others have reported an overall loss of core histones in senescence [36,37]. It is currently not clear how these findings can be reconciled. Therefore, the apparent relaxation of heterochromatin in LADs and pericentromeres for the time being should be taken as being relative to the rest of the senescent cell genome, as we do not yet know the absolute values of LAD heterochromatin compaction between young and senescent cells.

Here we report similar chromatin changes taking place during the normal aging of two mouse tissues (liver, skeletal muscle) as we have previously documented in senescent cells in vitro. In addition, we have added two new analytical methods to those used previously: assessment of the sensitivity of chromatin to nuclease digestion in intact, permeabilized nuclei, and a quantification of the overall polyadenylated mRNA content per cell. By both measures the overall level of chromatin compactness increases with aging: nuclei become more resistant to nuclease (Figure 1), and the total levels of mRNA decrease (Figure 2). These trends are consistent with our previous findings of age-associated increases in the levels of a heterochromatin associated protein (macro H2A) in the same tissues [26].

Most notably, we have shown here that the expression of both RTEs and SEs increases with age in mouse tissues. As in human cells, we found increased expression of both LINEs and SINEs. The mouse genome is more permissive for retrotransposition than the human genome, and several families of LTR retrotransposons are known to be active in the mouse. In agreement, we found age-associated increases in the expression of the LTR RTE MusD. Importantly, we have also found that increased transcription of L1 and MusD elements is associated with significant increases in their copy numbers, which is indicative of active transposition. In the case of L1, the primers we used detect approximately 5,000 (mostly evolutionarily recent) elements; hence, a 2-fold increase in copy number detected with these primers would be consistent with approximately 5,000 new insertions throughout the mouse genome. Finally, as in human cells, the most pronounced changes, both in terms of relatively early onset as well as a large magnitude, were found in the expression of pericentromeric satellite sequences. Although these elements do not transpose, they are known to be heavily heterochromatinized. The magnitude of the changes seen in satellites suggests that aging in the mouse affects globally the compaction of constitutive heterochromatin.

The changes that affect RTEs and SEs in mouse tissues very closely resemble our recent data obtained in a cell culture model of replicative cellular senescence [25]. It is unlikely, however, that the *in vivo* activation of RTEs can be attributed wholly to senescent cells. First, senescent cells in normal tissues are quite rare even at advanced ages. Second, skeletal muscle is a tissue with one of the lowest reported frequencies of senescent cells, whose levels do not appreciably increase with aging [38-40]. Skeletal muscle is composed predominantly (>90%) of terminally differentiated, postmitotic cells (myocytes). One interpretation of our findings is that chromatin changes and RTE mobilization are not a unique feature of cellular senescence, but can also occur in chronologically aging postmitotic tissues. In other examples, the *S. cerevisiae* LTR RTE Ty1 is mobilized during chronological aging of this species [41], and several *D. melanogaster* LINE and LTR RTEs become active in aging neurons [42]. Hence, the age-associated “loosening” of RTEs appears to be conserved and can be found in several postmitotic cell types. Another interpretation is that postmitotic cells can acquire some phenotypes typically associated with cellular senescence [43].

It has been suggested that transposition may be beneficial by promoting new patterns of gene expression that could lead to evolutionary bursts [11]. It has also been argued that somatic retrotransposition is likely to be overwhelmingly deleterious [44]. The destabilizing effects of RTEs are not solely due to insertional mutagenesis, as retrotransposition is accompanied by many incomplete events that result in DNA double strand breaks [12-14]. In turn, the transcription of RTEs and SEs is activated by DNA damage and a variety of stresses, creating a strong potential for positive feedback loops of increasing genome instability [45-47].

Until recently retrotransposition in the soma was thought to be quite rare; however, as methods to detect these events have improved, so have reports of their occurrence. Somatic L1 retrotransposition has been noted in mouse neural progenitor cells and in human adult brain tissue [17,19]. Here we extend these findings by showing that retrotransposition increases strongly with advanced age. Retrotransposition occurs frequently during human cancer development, but to what extent this is causal is still debated [22]. Our data here extend these reports by showing that RTE activation and mobilization also occurs in naturally occurring cancers in the mouse. This finding should pave the way to studying the functional role of retrotransposition in the development of cancer using a variety of mouse genetic models.

The most important finding presented here is the apparent activation of retrotransposition during normal aging. We suggest that somatic retrotransposition is a potentially important aging process that can affect many tissues, and that the mobilization of RTEs is likely to be a significant contributor to the progressive dysfunction of aging cells [48]. Reverse transcriptase, the cornerstone of the retrotransposition process, can be pharmacologically inhibited. Several antiviral drugs widely used in the treatment of HIV and HBV infections have been found to inhibit the L1 reverse transcriptase [49]. Given the large amount of research in this area, new compounds directed specifically towards the L1 enzyme could likely be developed.

## METHODS

### Tissue specimens

Mouse tissues of strain C57BL/6 were obtained from the NIA Aged Rodent Tissue Bank (www.nia.nih.gov/research/dab/aged-rodent-tissue-bank-handbook), or collected in-house from mice obtained from the NIA Aged Rodent Colonies (www.nia.nih.gov/research/dab/aged-rodent-colonies-handbook).

### Nuclease sensitivity

The DNase I sensitivity assay is a long-standing and well-established method to assess chromatin compaction in intact nuclei [50]. Nuclei were isolated from intact frozen tissues. All procedures, unless indicated otherwise, were performed in the cold (4°C or on ice). For liver, the tissue was homogenized in a Dounce homogenizer in buffer A1 (15 mM HEPES pH 7.5, 300 mM sucrose, 60 mM KCl, 15 mM NaCl, 2 mM EDTA, 0.5 mM EGTA, 0.15 mM spermine, 0.5 mM spermidine, 14 mM mercaptoethanol, and protease inhibitors) by douncing 5x with the loose pestle and 3x with the tight pestle. The homogenate was centrifuged at 1800 rpm for 5 min. The pellet was resuspended in buffer A2 (A1 + 0.5% NP-40) and gently layered on top of a cushion of buffer B (A1 made with 60% sucrose). Nuclei were collected by centrifugation at 2500 rpm. Nuclei were washed once in DNase I buffer (100 mM NaCl, 50 mM Tris/HCl pH 8.0, 3 mM MgCl_2_, 0.15 mM spermine, 0.5 mM spermindine) and resuspended in the same buffer to a final concentration of 5×10^6^ nuclei/ml. 5×10^5^ nuclei were placed in an ice water bath and DNase I (0.5-2.5 units) was added to the nuclei with CaCl_2_ to a final concentration of 1 mM. The tubes were placed in a room temperature water bath for 10 min, and then moved back to the ice water bath for 30 sec. The reaction was stopped by adding EDTA to a final concentration of 10 mM.

For muscle, the tissue was finely minced in homogenization buffer (10 mM HEPES pH 7.5, 10 mM MgCl_2_, 60 mM KCl, 300 mM sucrose, 0.1 mM EDTA, 0.1 % Triton X-100 and protease inhibitors), homogenized using a PowerGen 125 homogenizer (Fisher Scientific) set on 3 for 20 sec, then placed on ice for 30 sec. The homogenization and cooling procedure was repeated 2 additional times. The sample was then centrifuged at 20x g for 5 min at 4°C to collect myofibrils and debris. The supernatant was carefully removed and saved. The pellet was resuspended in homogenization buffer and homogenized and centrifuged as described above. This process was repeated again for a total of 3 homogenization and centrifugation steps for each sample. The supernatants were combined and the myonuclei were collected by centrifugation at 450x g for 10 min at 4°C. The myonuclei were washed in DNase I buffer and resuspended at 5×10^6^ nuclei/ml in DNase I buffer.

For analysis of the DNA size distribution, DNase I-treated nuclei were digested overnight with 70 μg of proteinase K at 37°C, the DNA was extracted with phenol:chloroform:isoamyl alcohol (25:24:1), followed by precipitation with 70% ethanol and 100 mM ammonium acetate pH 5.3. The DNA fragments were separated by electrophoresis on a 0.8% agarose gel run in 1x TAE (40 mM Tris-acetate, 1 mM EDTA) containing 1x SYBR Safe dye (Invitrogen) in both the gel and the running buffer. The gel was imaged using a Typhoon 9410 Variable Mode Imager and the lanes were quantified using ImageJ open source software (www.rsbweb.nih.gov/ij/).

### Comet assay

As an alternative method of analysis DNase I-treated nuclei were subjected to single cell electrophoresis, known as the Comet assay, which has been developed and extensively optimized as a highly sensitive method to quantify a variety of DNA lesions [51]. Nuclei were isolated and treated with DNase I as described above. Nuclei were then suspended in 1% low temperature gelling agarose in 1x Dulbecco's phosphate buffered saline (PBS) without Ca^2+^ and Mg^2+^ and containing 10 mM EDTA and placed on flare assay slides (R&D Systems). The agarose was allowed to harden for 10 min at 4°C. The slides were incubated for 1 hr in lysis buffer solution (Trevigen), and then electrophoresed for 20 min at 1 V/cm in neutral electrophoresis buffer (100 mM Tris pH 9.0, 300 mM sodium acetate, 10 mM EDTA). The DNA was precipitated *in situ* by incubating the slides in 70% ethanol, 1 M ammonium acetate for 30 min, followed by a 30 min incubation in 70% ethanol. Slides were dried for 15 min at 37°C, stained with 1x SYBR Gold for 30 min, dried at 37°C, mounted and visualized using a Zeiss LSM 710 confocal microscope. Images were analyzed using either the open source software CellProfiler [52] or ImageJ software. Comet tail length, moment, and percent DNA in the head and tail were calculated for each nucleus. Statistical significance was determined using Student's t-test.

### Immuno-FISH

Tissues were embedded in Tissue-Tek optimal cutting temperature compound (OCT; Sakura Finetek) at the time of harvest and stored at −80 °C. 8 mm tissue sections were cut using a cryomicrotome (Leica CM3050S) at −19°C. 4% paraformaldehyde and 0.5% Triton X-100 in PBS, pre-warmed to 37 °C, were used to fix the samples for 20 min at room temperature. Samples were then washed in PBS. Fluorescence *in situ* hybridization (FISH) with oligo-dT-Cy3 was performed as described previously by Piacentini et al. [53]. Briefly, to detect polyA RNA, liver sections were incubated for 30 min at 37°C in prehybridization buffer (2x SSC, 20% formamide, 0.2% BSA, 1 mg/ml yeast tRNA). For hybridization, the sections were transferred to a humidified chamber and incubated in 20 μl of hybridization buffer (prehybridization buffer plus 10% dextran sulfate) supplemented with 2 pmol/μl oligo-dT(22) fluorescently end-labeled with Cy3 (Integrated DNA Technologies). The samples were hybridized for 3 h at 37°C and then washed twice for 5 min in 2x SSC, 20% formamide (37°C), then 2x SSC (37°C), 1x SSC (room temperature), and finally in PBS (room temperature). The specimens were then incubated with anti-E-Cadherin antibodies (BD Transduction Laboratories, Cat. No. 610181) diluted in blocking solution (4% bovine serum albumin (BSA; fraction V, Thermo Fisher), 2% donkey serum, and 0.1% Triton X-100 in PBS) for 2 hr at room temperature with rocking in a humidified chamber. The specimens were then washed twice in PBS and incubated with secondary antibodies (AlexaFluor 647, Invitrogen) for 1 hr at room temperature with rocking in a dark humidified chamber. DNA was counter-stained with DAPI and the slides were mounted in anti-fade medium (Invitrogen).

### Quantitative immunofluorescence

Immuno-FISH images were acquired using a Zeiss LSM 710 Confocal Laser Scanning Microscope. All microscope settings were set to collect images below saturation and were kept constant for all images taken in one experiment, as previously described [26]. All images were collected at 16-bit resolution in order to maximize the dynamic range of the detected intensities. Images were analyzed with the open source software CellProfiler [52] and CellProfiler Analyst [54]. ImageJ was used to convert the ZEN format files generated by the Zeiss software into TIFF files recognized by the CellProfiler software. The analysis was automated by the development of Cell Profiler pipelines (available on request) as previously described [55], allowing the processing of large numbers of images and recording all values in databases. For each database values from at least 500 cells were compiled. Nuclei were defined using the DAPI channel and cell boundaries were defined using E-cadherin-647 nm channel. Statistical significance (p value) was assessed using the two-tailed Student's test (t-test).

### Design of PCR primers

All primers used in this study are listed in Table S1. For expression analysis of LINE L1, MusD/ETn and pericentromeric γ-satellite sequences (MSAT) we used primers described by Changolkar et al. [56]. Primers for the SINE elements B1 and B2 were designed using the consensus sequence from Repbase (Genetic Information Research Institute, www.girinst.org/repbase/index.html) and Primer-Blast software (www.ncbi.nlm.nih.gov/tools/primer-blast/). Primers to detect specific subfamilies of RTEs were designed using Primer-Blast. Primer sequences were analyzed using the UCSC genome browser *in silico* PCR tool (genome.ucsc.edu/cgi-bin/hgPcr) to determine the number of genomic elements that contribute to the amplification products, as described [25].

### Quantitative real-time PCR

qPCR of both and RNA and DNA was performed using the SYBR Green system (Applied Biosystems) on the ABI 7900 Fast Sequence Detection instrument (Life Technologies), according to the manufacturer's specifications, with the exception of the multiplex assay to detect transposition events (relative RTE copy number in the genome), which was performed using the TaqMan Gene Expression system (Applied Biosystems), as originally described by Coufal et al. [17] and subsequently modified as described by De Cecco et al. [25]. The forward and reverse primers for both MusD/ETn and 5S were used at 400 nM final concentration. Probes for both MusD/ETn and 5S were used at 187.5 nM final concentration. L1 primers and probe final concentrations were 300 nM and 130 nM, respectively.

For qPCR of RNA, total RNA was harvested from tissues using Trizol reagent (Invitrogen) according to the manufacturer's instructions. A quantity of 1 μg of total RNA was transcribed into cDNA in 50 μL reactions using the TaqMan kit (Applied Biosystems), according to the manufacturer's protocol. A volume of 0.1-1.5 μL of this reaction was used in subsequent qPCR reactions. GAPDH was used as the normalization control. For the measurement RTE and SE transcription, prior to the synthesis of cDNA, total RNA was exhaustively digested with RNase-free DNase (Qiagen), and further cleaned up on RNeasy columns (Qiagen), as described [25]. The effectiveness of the DNase digestion was assessed using controls that omitted reverse transcriptase. All the data presented here were carefully controlled in this manner; all samples that showed DNA contamination were either re-digested and re-purified or discarded.

To prepare genomic DNA for qPCR, a tissue sample of 25-30 mg was finely minced and incubated with 500 μl of lysis solution (10 mM Tris-HCl pH 8.0, 1 mM EDTA, 1.5% SDS, 200 μg proteinase K) overnight at 65°C with constant agitation. The samples were dounced 5x with a sterile pestle and digestion was continued for 5 hr. After cooling down to room temperature the samples were extracted with 1volume of phenol (vortexed for 2 min), followed by another extraction with phenol-chloroform-isoamyl alcohol (25:24:1) and finally ethanol precipitation. Pellets were resuspended, digested for 2 hr with RNase A, and sonicated to a fragment size of 300-600 bp. A final purification with phenol-chloroform-isoamyl alcohol and ethanol precipitation was performed, the DNA was resuspended in 10 mM Tris-HCl pH 8.0, 1 mM EDTA, and quantified using the Qubit HS DNA assay. For preparation of DNA from formalin fixed tissues, a tissue sample of 25-30 mg was first washed 3x in PBS and subsequently finely minced. The lysis solution was substituted with de-crosslinking solution (10 mM Tris-HCl pH 8.0, 1 mM EDTA, 1.5% SDS, 0.2 M NaCl, 200 μg proteinase K) but otherwise the protocol described above was followed.

### RNA-seq

30-50 mg of liver was used for RNA extraction. Polyadenylated mRNA was prepared using two consecutive purifications with oligo-dT magnetic beads, following the mRNA Direct Dynabeads Kit protocol provided by the manufacturer (Invitrogen). The eluted mRNA was quantified using the Qubit 2.0 RNA HS Assay Kit (Invitrogen). 200 ng of mRNA was fragmented using the RNA Fragmentation Kit (Ambion) in a thermocycler at 70°C for 5 min. Fragmented mRNA was precipitated with isopropanol and glycogen (Ambion), resuspended in RNAse-free water, and reverse transcribed using the SuperScript III First Strand Kit (Invitrogen). A non strand-specific second strand DNA synthesis was performed using the Second Strand Buffer (New England Biolabs), DNA polymerase I and RNAse H in a pre-cooled thermocycler for 2.5 hours at 16°C. 20 ng of double-stranded cDNA per sample was end repaired with the End-It DNA End Repair Kit (Epicentre) according to the provided protocol. DNA was purified using Agencourt AMPure XP Paramagnetic Beads (Beckman Coulter), and eluted in molecular grade water. dATP was added to the DNA ends [57], and after another DNA purification, pre-annealed adapters were ligated to each sample [58]. 10 cycles of PCR amplification were performed using Phusion High-Fidelity DNA Polymerase (New England Biolabs), and the libraries were agarose gel purified retaining fragments in the range of 200-500 base pairs. RNAseq libraries were sequenced on an Illumina HighSeq 2000 instrument by the Brown University Genomics Core to generate single-end 50 nucleotide reads. The sequencing data were uploaded to the Galaxy platform (main.g2.bx.psu.edu/) [59-61] and mapped with TopHat against the mouse genome (mm10) [62]. Cufflinks was used to estimate gene transcript abundance in reads per kilobase per million mapped reads (RPKM) and Cuffdiff was used for analysis for differential gene transcript expression [63].

### Computational analysis of differential repetitive element enrichment

Computational analysis of repetitive element enrichment was performed on mouse liver RNA-seq datasets that were generated in triplicate biological replicates from 5, 24 and 36 month old animals. This analysis was performed using a new method, RepEnrich [27], which is an extension of previous strategies to estimate the enrichment of repetitive elements using high-throughput sequencing data [64]. In contrast to previous methods, RepEnrich does not exclude any mapping reads in the estimation of repetitive element enrichment. In brief, RNA-seq reads were first mapped to the mouse genome (mm9) using Bowtie1 with options that only allow unique alignments [65]. The unique mapping reads contained within the alignment file (BAM) were sorted and tested for overlap to mm9 RepeatMasker (www.repeatmasker.org/) annotated repetitive elements. Next, RNA-seq reads mapping to multiple locations were mapped to repetitive element pseudo-genomes that represent all RepeatMasker annotated genomic instances of repeat sub-families in a manner similar to Day et. al [64]. If a read mapped to a single repeat sub-family pseudo-genome it was counted once within that repeat sub-family, while reads mapping to multiple repeat sub-family pseudo-genomes were assigned a value of 1/(number of repeat sub-families aligned). Counts for the unique mapping and multi-mapping reads were then added to compute the repeat element sub-family enrichment. The final count data was rounded to the nearest integer for the follow-up differential enrichment analysis.

To conduct differential analysis of repetitive element enrichment between the age groups the repetitive element sub-family count data for the three replicates from 5, 24 and 36 month animals was first normalized using the trimmed mean of M-values (TMM) normalization method [66], and manually inputted library sizes. The library sizes were determined using the total mapping reads from the same experimental samples mapped to the mm9 mouse genome using Tophat with the default parameters (multi-map reads and reads mapping to splice junctions are included in the estimate of library size). Differential enrichment was then tested by fitting a multi-factor generalized linear model (GLM) using EdgeR [67]; each of the experimental pair-wise comparisons was denoted as a separate contrast (see EdgeR tutorial). Although traditionally used to conduct differential analysis of gene expression count data from RNA-seq experiments, such models are now more widely applied to diverse types of genomic count data that can be fitted with negative binomial (NB) distributions [68]. We also used EdgeR to compute the log2 counts per million (CPM) for each sample and calculated the average log2 CPM for 5, 24 and 36 month animals. The p values that were computed from the EdgeR GLM represent the statistical test for differential enrichment of the repetitive element sub-families for each experimental comparison. The p values were corrected using an FDR correction that corrects for multiple hypothesis testing according to Storey et al. [69].

## SUPPLEMENTAL INFORMATION


