# Supplementary material for: Transposable elements become active and mobile in the genomes of aging mammalian somatic tissues
Source: Aging (Albany NY). 2013 Dec 7;5(12):867–83. doi: 10.18632/aging.100621 (PMC3883704; doi:10.18632/aging.100621)
Supplement: Supplementary file 1 [file aging-05-867-s001.pdf]

SUPPLEMENTAL INFORMATION

Additional Experimental Information and Statistical Treatment for Figures 1, 2, 4, 6 and 7

Figure 1. DNase I sensitivity of chromatin in intact nuclei. (**A**) Four animals per age group were analyzed in 3 independent experiments. A representative experiment is shown. Differences between young and old animals were significant for 1.0 units of DNase I at $p \leq 0.01$, and for 0.5, 1.5 and 2.0 units of DNase I at $p \leq 0.05$; 2.5 units were not significant. (**B**) A minimum of 50 nuclei were visualized for each treatment group. For liver, 4, 2 and 4 animals were analyzed per 5, 24 and 36 month age groups, respectively, in 4 separate experiments. A representative experiment is shown. Differences between 36 month old and either 24 or 5 month old animals were significant at $p \leq 0.01$ for both 0.5 and 0.75 units of DNase I. The differences between 5 month old and 24 month old animals were not significant. For muscle 3 animals were analyzed in each age group in 3 separate experiments. A representative experiment is shown. Differences between 36 month old and either 24 or 5 month old animals were significant at $p \leq 0.01$ for 1 unit of DNase I. The difference between 5 month old and 24 month old animals was significant at $p \leq 0.05$ for 1 unit of DNase I. All p values were calculated using the 2-tailed Student's t test.

Figure 2. Total mRNA expression in aging liver. (**A**) 3 animals were used for each age group. All samples in one experiment were processed in parallel. Differences between all comparisons were significant at $p \leq 0.01$. The same results were obtained whether the RNA yields were normalized to tissue weight or genomic DNA. Repeated independent experiments showed that this effect was statistically reproducible. (**B**) At least 500 cells were imaged for each sample. 3 animals were used for each age group. The difference was significant at $p \leq 0.05$. All p values were calculated using the 2-tailed Student's t test.

Figure 4. qPCR analysis of RNA expression of representative RTEs and SEs. (**A, B**) 5 animals were used per age group. Equivalent amounts of RNAs were pooled for each age group and assayed in triplicate. All samples were run in parallel, and a minimum of 3 independent experiments were performed. Means and standard deviations of 3 independent experiments are shown. Data were additionally normalized to the 5 month value for each element (shown as 1.0). For liver (panel A) differences between 5 and 24 months were significant at $p \leq 0.01$ for MusD, B1 and MSAT, and at $p \leq 0.05$ for B2; between 5 and 36 months at $p \leq 0.01$ for L1, MusD, B1, B2 and MSAT, and between 24 and 36 months at $p \leq 0.01$ for L1, B1 and at $p \leq 0.05$ for B2. For muscle (panel B) differences between 5 and 24 months were significant at $p \leq 0.01$ for L1, MusD, B1, B2 and MSAT, between 5 and 36 months at $p \leq 0.01$ for L1, MusD, B1, B2 and MSAT, and between 24 and 36 months at $p \leq 0.01$ for MusD, B1 and B2, and at $p \leq 0.05$ for MSAT.

Figure 6. qPCR analysis of DNA to assess RTE genome copy number. Experiments were performed, analyzed and presented as in Figure 4 except: 1) DNA was quantified instead of RNA; 2) The TaqMan system was used instead of the SYBR green system; 3) Internal normalization was to 5S rRNA genes. All differences between 5 and 36 months and 24 and 36 months were significant at $p \leq 0.01$, except muscle MusD, which was at $p \leq 0.05$. None of the differences between 5 and 24 months were significant. All p values were calculated using the 2-tailed Student's t test.

Figure 7. qPCR analysis of DNA to assess RTE genome copy number in spontaneously occurring tumors. In (**A**) 3 female mice that were aged without any interventions were analyzed. Mouse 729 died naturally at 28 months of age. Mouse 782 was sacrificed in apparent normal health at 30 months of age. Mouse 868 died naturally at 24 months of age. Tumor tissues were found at time of autopsy and preserved in formalin. (**B**) 4 male mice that were aged without any interventions were analyzed. Mouse 765 died naturally at 24 months of age. Mice 1362, 1365 and 1413 were part of a larger experiment in which animals were sacrificed at 24 months of age in apparent normal health for tissue samples. The tumor tissues used here were found at the time of autopsy and preserved in formalin.

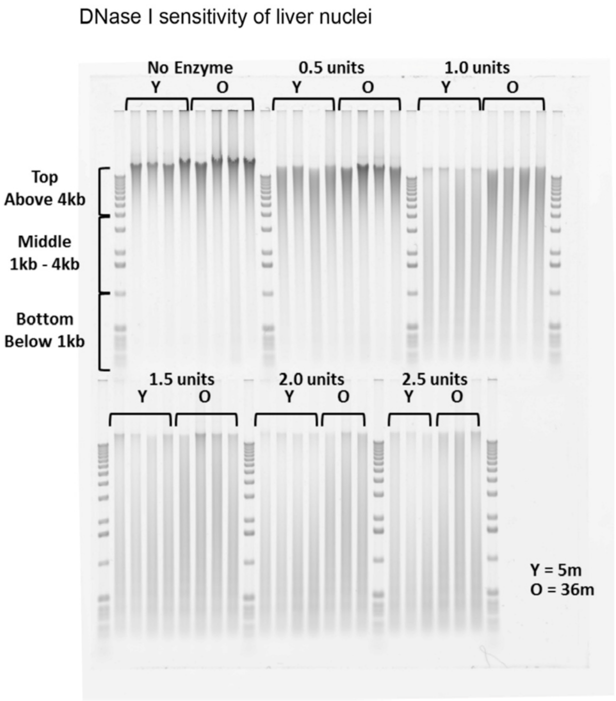

Supplemental Figure 1. Gel electrophoresis of liver nuclei digested with increasing concentrations of DNase I. A representative experiment is shown. All samples were processed in parallel. Y, 5 month old; O, 36 month old. A compilation and graphical representation of these experiments is shown in Figure 1A.

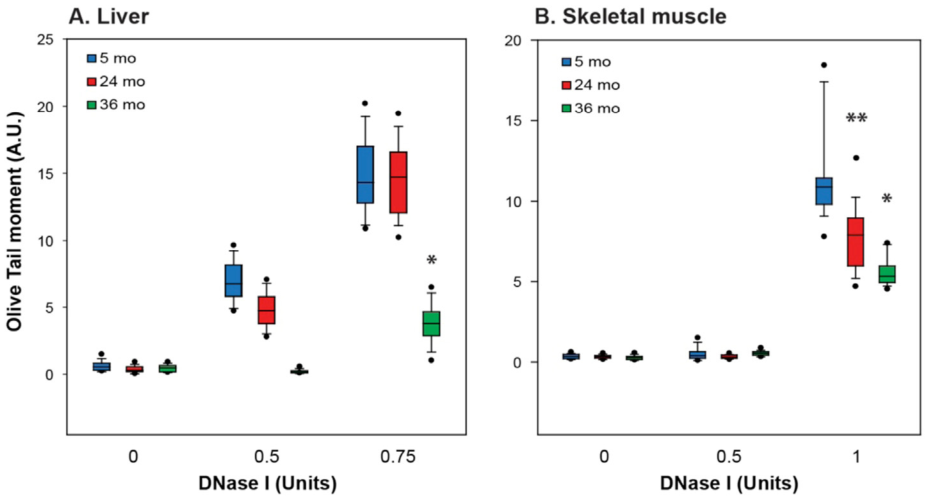

Supplemental Figure 2. Comet assays quantified using the Olive tail moment. Data from the same experiments as shown in Figure 1B were used, but were quantified using a different method. The Olive tail moment is defined as the mean signal of the tail minus the mean signal of the head, times the percentage of DNA in the tail, divided by 100. The results of this method of analysis are completely consistent with the data shown in Figure 1B. Data are represented as box plots, where the box shows the median 50% (ranging from 25% to 75% and the line being the median), the whiskers the 95% and 5% range, and the dots the top and bottom 5% of the values. (**A**) Liver. Differences between 36 month old and either 24 or 5 month old animals were significant at $p \leq 0.01$ for both 0.5 and 0.75 units of DNase I. The differences between 5 month old and 24 month old animals were not significant at either 0.5 and 0.75 units of DNase I. (**B**) Skeletal muscle. Differences between 36 month old and either 24 or 5 month old animals were significant at $p \leq 0.01$ for 1 unit of DNase I. The difference between 5 month old and 24 month old animals was significant at $p \leq 0.05$ for 1 unit of DNase I. All p values were calculated using the 2-tailed Student's t test.

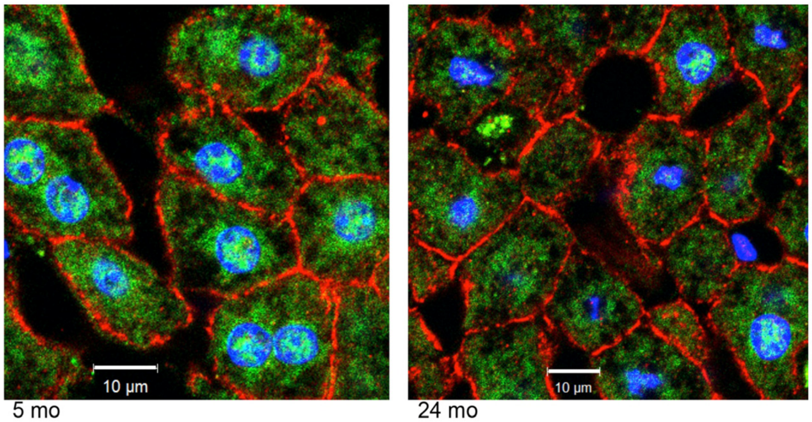

Supplemental Figure 3. Representative images of oligo-dT immuno-FISH staining of liver sections. The quantification of these data is shown in Figure 2B. Left panel, 5 month old liver; right panel, 24 month old liver. Nuclei were stained with DAPI (blue). E-cadherin staining (red) highlights cellular membranes. Intracellular mRNA was detected using Cy3-oligo-dT probes, and the signal from this staining is shown in the green channel. Each age group was represented by 3 animals, and 3 independent experiments were performed. Images were acquired at 63x magnification with an oil immersion objective using a Zeiss 710 confocal microscope. Scale bar = 10 μm.

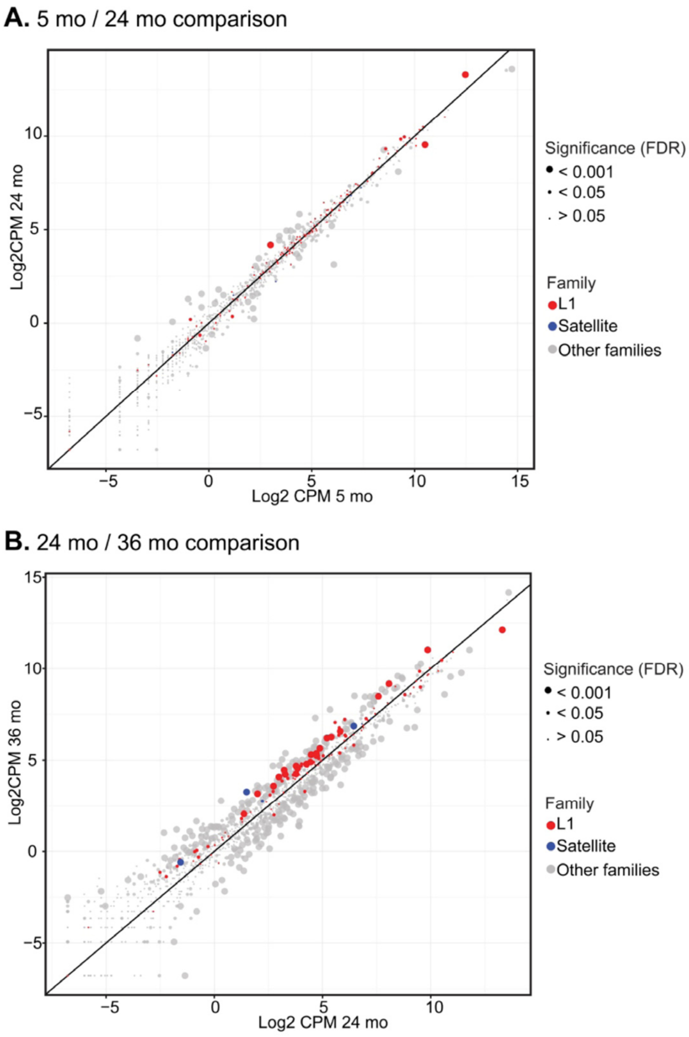

Supplemental Figure 4. Relative enrichment of RTE sequences in RNA-seq databases. Sequence tags mapping to repetitive elements were scored with RepEnrich software for liver samples of 5, 24 and 36 month old mice (3 animals per group). The data were processed and are displayed as in Figure 3A, with the exception that the 5-24 month comparison (**A**) and the 24-36 month comparison (**B**) are shown here. As in Figure 3A, each point represents a sub-family, and the points falling above the diagonal are enriched in old animals. The size of the points indicates the FDR corrected significance levels. L1 and SE sub-families are shown highlighted in color. Note that the majority of the changes take place in the 24 to 36 month interval. (CPM, counts per million).

Supplemental Table 1. List of primers

Primer Name – TaqMan assays	Sequence
5S probe	AGGGTCGGGCCTGG-6FAM
5SF	CTCGTCTGATCTCGGAAGCTAAG
5SR	GCGGTCTCCCATCCAAGTAC
LINE probe	TGGTTCGAACACCAGATATCTG-TET
LINEF	TGAGTGGAACACAACTTCTGC
LINER	CAGGCAAGCTCTCTTCTTGC
MusD/ETn probe	AGTGCAGGAGCAGTTAGAAGC-HEX
MusD/ETn F	ATAGAGGCCGCTTCTTTGC
MusD/ETn R	TGAGACTCCACCAAATGTCC

Primer Name – RTE expression	Sequence
L15'UTRF	CTGCCTTGCAAGAAGAGAGC
L15'UTRR	AGTGCTGCGTTCTGATGATG
LINEF	TGAGTGGAACACAACTTCTGC
LINER	CAGGCAAGCTCTCTTCTTGC
L1ORF1F	AGATCTGGAACCATAGATG
L1ORF1R	AATCCAGGACACAATGAGAA
L13'UTRF	CCAGCAAACACAGAAGTGGATGCTCA
L13'UTRR	TTTGCAAGTCCAATGGGCCTCTCT
MLV5F	TTCCCAATAAAGCCTCTTGC
MLV5R	AGACCCTCCCAAGGATCAGC
LINEF	TGAGTGGAACACAACTTCTGC
LINER	CAGGCAAGCTCTCTTCTTGC
IAPLTR41F	CCTCTCCACGGGTCTTGAAC
IAPLTR41R	TAGGGACCTCCGCTGATTGA
IAPLTR42F	CCACGGGTCTTGAACCTGAG
IAPLTR42R	CTCTAGGGACCTCCGCTGAT
ETnERV3I1F	CAGGAGGGCAAGATGCTCAA
ETnERV3I1R	CAAGCTTCTCTGAGGCTGCT
ETnERV3I2F	CCTTCGAACAGGGACACCAG
ETnERV3I2R	GCGGTTGACGAGGTCCTATC
